# Implementing a Preoperative Holding Area: Feasibility and Early Effects on First-Case Orthopaedic Theatre Starts in a Major Trauma Centre

**DOI:** 10.7759/cureus.96292

**Published:** 2025-11-07

**Authors:** Duy Anh Do, Himani Murdeshwar, Brogan Jamson, Alan Norrish

**Affiliations:** 1 Department of Trauma and Orthopaedics, Nottingham University Hospitals NHS Trust, Nottingham, GBR; 2 Academic Unit of Injury, Recovery and Inflammation Sciences, School of Medicine, University of Nottingham, Nottingham, GBR

**Keywords:** healthcare process efficiency, major trauma centre, operative delay, theatre efficiency, theatre productivity, trauma and orthopaedic

## Abstract

Background

Timely first-case starts underpin theatre productivity and reduce downstream delays. We evaluated whether introducing a proximal preoperative holding area improved first-case start performance across four orthopaedic theatres in a major trauma centre (MTC).

Methods

We conducted a prospective quality-improvement project (QIP) comparing a 2-week pre-intervention baseline with a 2-week post-intervention assessment period. The paediatric anaesthesia recovery bay was repurposed as a preoperative holding area for eligible adult first-case patients when available. Paediatric cases and cancelled lists were excluded. Operational timestamps were extracted from operating room management software and converted to minutes after 08:00. Primary outcomes were first-case send-for time and anaesthetic start time. Group comparisons used two-sided Welch’s *t*-tests with mean differences and 95% CIs. On-time performance was defined *a priori* as anaesthesia commencing ≤09:10 (≤70 minutes after 08:00) and compared using ORs with 95% CIs.

Results

We analysed 110 operating lists: 40 pre-intervention and 70 post-intervention, of which 35 used the holding bay. Relative to baseline, lists that used the holding bay demonstrated an earlier send-for by 8.2 minutes (mean 25.4 → 17.2 minutes after 08:00; mean difference -8.2; 95% CI -15.8 to -0.6; *p*=0.035), indicating significantly improved patient readiness. The mean anaesthetic start moved earlier by 2.3 minutes (79.2 → 76.9 minutes after 08:00; mean difference -2.29; 95% CI -10.1 to +5.6; *p*=0.56), a non-significant change. On-time starts increased from 35.0% (14/40) pre-intervention to 42.9% (15/35) with holding-bay use (OR 1.39; 95% CI 0.55-3.54). Analyses including all post-intervention lists, irrespective of holding-bay use, showed similar but attenuated effects.

Conclusions

Implementation of a preoperative holding area significantly advanced preoperative readiness and modestly shifted first-case anaesthetic starts earlier. The translation from earlier send-for to earlier induction was constrained by downstream operational factors. Characterisation of these constraints is essential to optimise upstream gains. Coupling dependable access to a dedicated holding space with a bundle, including pre-identification and pre-assessment of a “Golden Patient” and punctual multidisciplinary briefs with named handover, offers a credible route to consistently on-time first-case starts and improved theatre productivity.

## Introduction

Queen’s Medical Centre (QMC), part of Nottingham University Hospitals NHS Trust, is the designated Major Trauma Centre (MTC) for the East Midlands and provides emergency and specialist care to a regional population of approximately four million [1]. The QMC campus incorporates the ED, the MTC, and Nottingham Children’s Hospital, and functions as the Trust’s principal emergency care site within one of the largest acute NHS trusts in England [2]. The scale and acuity of this catchment place sustained pressure on surgical services, highlighting the operational importance of reliable on-time first-case starts in orthopaedics.

National improvement programmes, including Getting It Right First Time (GIRFT) and The Productive Operating Theatre (TPOT), advocate standardising preoperative pathways, optimising list construction, and refining team processes to increase theatre productivity and reduce cancellations, under-utilisation, and overruns [3-5]. Late first-case starts are a recognised driver of downstream delays: they compress anaesthetic and surgical windows, diminish list utilisation, and increase the likelihood of end-of-day overruns and case cancellations [6,7]. Within QMC, observational data and stakeholder feedback suggested that variability in patient readiness immediately before the morning brief contributed materially to these delays. In other centres, first-case on-time starts (FCOTS) also serve as key theatre efficiency metrics, and improving punctuality can deliver substantial financial savings [7-10].

To address this, we implemented a pragmatic preoperative holding area adjacent to theatres to ensure that the first patient was fully prepared before the morning safety brief. This has been shown in other trusts to reduce theatre downtime by 90% and halve the time between procedures, leading to an increase in the number of in-hours procedures completed [11]. Owing to space constraints, the paediatric recovery bay was repurposed for this function when available. Its proximity to the operating theatres, together with a fully equipped environment, including essential monitors and capacity to carry out the pre-anaesthetic checklist, made this an ideal location. This service change was a low-cost, workflow-focused intervention intended to reduce non-clinical delays without altering case mix, staffing, or theatre capacity.

We conducted a prospective quality-improvement project (QIP) to evaluate the impact of this intervention on first-case timeliness. Two audit cycles were carried out using a standard Plan-Do-Study-Act (PDSA) model to guide the development of this project. The primary aim was to determine whether a preoperative holding bay improves FCOTS. Secondary objectives were to (i) increase the proportion of orthopaedic lists achieving an on-time start, (ii) shorten pre-induction and sign-in intervals, and (iii) reduce variation in first-case start times. In parallel, we sought to characterise residual bottlenecks such as patient flow, porter availability, and preoperative documentation to inform subsequent cycles of improvement. This paper reports the design, implementation, and outcomes of the holding-area intervention and outlines actionable next steps for sustaining and scaling theatre start-time reliability at QMC.

## Materials and methods

This prospective QIP evaluated four orthopaedic theatres at QMC and was registered as a service evaluation with Nottingham University Hospitals NHS Trust (registration no. 23-633C). Reporting follows SQUIRE 2.0 guidance [12]. The project focused on the operational reliability of first-case starts and was conducted under existing clinical governance arrangements; no changes to case mix, staffing, or capacity were introduced as part of the intervention.

All eligible first-case lists were included across two contiguous periods: a pre-intervention baseline taken over two weeks before the introduction of the intervention and a two-week post-intervention phase immediately after the intervention was initiated. Within the post-intervention phase, lists that used the holding bay were prospectively flagged for subgroup analysis. Paediatric cases, cancelled lists, out-of-hours procedures, and critical care admissions were excluded a priori. Operational timestamps were extracted from Bluespier Theatres (Bluespier International Ltd, London, UK) and handled as de-identified records; no patient-identifiable information was collected or processed. Patients with missing timestamps were excluded from the dataset.

A consensus was reached following discussions with key staff members (theatre scrub staff, theatre managers, anaesthetists, and surgeons) to repurpose the paediatric recovery bay, when available, as a preoperative holding bay for eligible adult orthopaedic first-case patients. At 08:00, first patients on the list were called down to the repurposed paediatric recovery bay. Four bays were kept available, with one nursing staff member from each theatre accompanying the patient with standard physiological monitoring while carrying out a pre-anaesthesia safety checklist. The intent was to ensure that the first patient was present, assessed, and ready in close proximity to theatres shortly after the team brief, thereby reducing non-clinical delays.

True first-case start time was defined as the anaesthetic start time of the listed first case. Therefore, the primary outcomes were (i) send-for time and (ii) anaesthetic start time. To facilitate analysis, clock times were transformed into minutes after the official list start time of 08:00 (e.g., 09:10 = 70 minutes). The secondary outcome was the proportion of lists achieving an on-time start, defined as anaesthesia commencing at or before 09:10 (≤70 minutes after 08:00). In addition, theatre-level Green/Yellow/Red distributions were derived according to the corresponding start-time thresholds used locally to categorise performance.

Descriptive statistics are reported as mean ± SD or as counts and percentages, as appropriate. For continuous outcomes, two-sided Welch’s t-tests (unequal variances) compared the pre-intervention period with (a) post-intervention lists that used the holding bay and (b) all post-intervention lists. Effects are presented as mean differences (post − pre) with 95% CIs and approximate two-sided p-values. For the binary on-time outcome, effect size is expressed as an OR with a 95% CI. All analyses were performed using Python 3.11 (Python Software Foundation, Wilmington, USA).

## Results

A total of 110 first-case orthopaedic lists were analysed: 40 in the pre-intervention period and 70 in the post-intervention period, of which 35 explicitly used the preoperative holding bay. Paediatric cases and cancelled lists were excluded, as prespecified.

For timing outcomes, lists that used the holding bay demonstrated a statistically significant improvement in patient “send-for” time. The mean send-for decreased by 8.2 minutes relative to baseline (from 25.4 to 17.2 minutes after 08:00; mean difference -8.2 minutes, 95% CI -15.8 to -0.6; p=0.035), indicating that first patients were available to the theatre suite earlier in the morning. In contrast, the mean anaesthetic start time improved modestly by 2.3 minutes (from 79.2 to 76.9 minutes after 08:00), but this difference was not statistically significant (mean difference -2.3 minutes, 95% CI -10.1 to +5.6; p=0.56) (Tables 1-2). When all post-intervention lists were considered irrespective of holding-bay use, the direction of effect was similar but attenuated: mean send-for improved by 6.7 minutes (95% CI -13.8 to +0.5; p=0.073) and mean anaesthetic start by 1.0 minute (95% CI -8.1 to +6.2; p=0.794) (Table 2). Together, these findings suggest that proximity-based readiness reliably brought patients to the threshold of anaesthesia earlier, but that downstream processes (e.g., induction and sign-in) constrained translation into a materially earlier anaesthetic start.

**Table 1:** Timing comparisons. Timing comparisons for the first case across all theatres: send-for and anaesthetic start times are expressed as minutes after 08:00 (mean values shown for the pre-intervention period (n=40) and the post-intervention period when the holding bay was used (n=35)). The table reports the mean difference (Post - Pre), t-based 95% CI, and two-sided p-value from Welch’s t-test (unequal variances). A negative mean difference indicates an earlier time post-intervention. Statistical significance: p < 0.05 (*). Asterisks denote significant p-values. HB: Holding bay.

Measure	Mean values (minutes)			P-value	95% CI
	Pre-intervention (n=40)	Post-intervention (HB used) (n=35)	Mean difference		
Send-for time (min)	25.4	17.2	-8.2	0.035 *	(-15.8, -0.6)
Anaesthetic start time (min)	79.2	76.9	-2.3	0.56	(-10.1, 5.6)

**Table 2:** Detailed inputs for statistical analysis (Welch t-test). Detailed inputs for statistical analysis (send-for and anaesthetic start), comparing Pre vs Post (holding-bay used) and Pre vs Post (all), which includes all theatres in the post-intervention period. For each comparison, the table lists n, mean (minutes after 08:00), SD, corresponding clock-time mean, mean difference (Post - Pre) with standard error (SE), Welch df, t-statistic, t-based 95% CI, and two-sided p-value from Welch’s t-test. Statistical significance: p < 0.05 (*). Asterisks denote significant p-values. HB: Holding bay; SE: Standard error; df: Welch degrees of freedom.

Comparison	Outcome	Pre (n=)	Pre mean (min)	Pre SD (min)	Pre mean (clock)	Post (n=)	Post mean (min)	Post SD (min)	Post mean (clock)	Mean diff (Post - Pre) (min)	SE of diff	df (Welch)	t-stat	95% CI for diff (min)	p-value
Pre vs Post (HB used)	Send-for (min after 08:00)	40	25.4	15.7	8:25	35	17.2	17.5	8:17	-8.2	3.88	68.8	-2.11	(-15.8, -0.6)	0.035 *
Pre vs Post (HB used)	Anaesthetic start (min after 08:00)	40	79.2	16.5	9:19	35	76.9	17.4	9:16	-2.3	3.93	70.4	-0.58	(-10.1, 5.6)	0.563
Pre vs Post (All)	Send-for (min after 08:00)	40	25.4	15.7	8:25	70	18.8	22.3	8:18	-6.7	3.67	101.2	-1.81	(-13.8, 0.5)	0.073
Pre vs Post (All)	Anaesthetic start (min after 08:00)	40	79.2	16.5	9:19	70	78.2	21.5	9:18	-1	3.66	99.1	-0.26	(-8.1, 6.2)	0.794

On-time performance, defined as anaesthesia commencing at or before 09:10 (≤70 minutes after 08:00), improved from 35.0% (14/40) at baseline to 42.9% (15/35) among lists that used the holding bay. The corresponding odds ratio for an on-time start was 1.39 (95% CI 0.55-3.54), indicating a favourable but imprecisely estimated effect that did not exclude the null. Examination of the theatre-level Green/Yellow/Red distribution echoed this pattern: relative to baseline, the proportion of “Green” starts increased (35.0% to 42.9%), while both “Yellow” (09:11-09:30) and “Red” (≥09:31) categories decreased (42.5% to 40.0% and 22.5% to 17.1%, respectively) (Table 3). Although these shifts are directionally consistent with improved start-time reliability, the study was not powered to detect small-to-moderate differences in categorical distributions.

**Table 3:** Green/Yellow/Red start-time overall distribution. Distribution of first-case anaesthetic start categories by cohort: Green ≤09:10 (on-time), Yellow 09:11-09:30, and Red ≥09:31; Pre (n=40) vs Post (HB used) (n=35). Rows show counts and percentages for each category. HB: Holding bay.

Anaesthetic start time	Pre-intervention (n=40)	Post-intervention (HB used) (n=35)	Pre (%)	Post (%)	OR	95% CI
Green ≤09:10	14	15	35	42.9	1.39	(0.55-3.54)
Yellow 09:11-09:30	17	14	42.5	40	0.9	(0.36-2.27)
Red ≥09:31	9	6	22.5	17.1	0.72	(0.23-2.25)

The principal quantitative signal, therefore, is an earlier send-for in the holding-bay subgroup (Table 1), supported by complete Welch t-test details in Table 2 (including standard deviations, standard errors, degrees of freedom, and t-statistics). The more modest and statistically non-significant changes in anaesthetic start times highlight residual bottlenecks after patient arrival in the holding area. These may include variability in staffing availability for induction, checklist completion, competing theatre set-up demands, and case-specific preparation requirements. Overall, the intervention appears to have improved pre-induction readiness and nudged punctuality towards the desired threshold, with scope for additional process optimisation to translate earlier availability into consistently earlier anaesthetic starts (Tables 1-3).

## Discussion

This quality improvement project tested a simple operational change: using a theatre-proximal preoperative holding area to determine whether first-case readiness could be advanced and whether that upstream gain would translate into earlier anaesthetic start times in a high-volume orthopaedic MTC. The results indicate feasibility and suggest potential benefit, rather than definitive evidence of improved theatre performance. Lists that used the holding bay sent for the first patient approximately eight minutes earlier than pre-intervention, and the proportion of on-time starts (≤09:10) increased by about eight percentage points. However, the mean anaesthetic start time showed only a non-significant improvement of about two minutes, and the CI crossed zero. Taken together, these findings indicate that the intervention advanced patient readiness but that downstream processes limited the translation of earlier availability into consistently earlier induction of anaesthesia.

That an earlier send-for only resulted in a modest improvement in anaesthetic start time is understandable in this operational context. The translation from earlier patient availability in the holding bay to earlier anaesthetic induction showed day-to-day variability, largely determined by downstream human and systemic factors. Residual bottlenecks included the timing and completeness of morning handovers, porter response times, variable ward-to-theatre distances, and intermittent gaps in full team presence at the morning brief. Other reasons reported in the literature for first-case delays include anaesthesia equipment-related issues, prolonged preoperative discussions, and incomplete patient evaluations [13]. In our series, the anaesthetic start moved only ~2.3 minutes earlier on average, despite a significant 8-minute improvement in send-for, consistent with bottlenecks after arrival (staff availability for induction, equipment/set-up, checklist completion).

External datasets highlight similar constraints. In large observational cohorts, the most common root causes of first-case delays include patient tardiness and surgeon tardiness, together accounting for the majority of late starts. One endoscopy-OR dataset reported patient tardiness in 44% and physician tardiness in 36% of first-case delays (mean 35 and 22 minutes late, respectively). These behavioural contributors directly attenuate the benefit of a prepared patient waiting in a holding bay [14]. Complementary studies of elective theatres likewise identify patient-related delays and surgeon availability among the top causes, with >50% of first cases starting late and median delays on the order of minutes that propagate to later cases [9]. Collectively, these data explain why a proximal holding bay reliably improves readiness but yields variable gains in induction time unless paired with behavioural reliability and operational discipline.

These results are generally consistent with national improvement programmes that emphasise disciplined preoperative pathways, reliable list starts, and minimisation of avoidable delays [3-5]. They suggest that a pragmatic “bundle” focused on execution could convert earlier readiness into reliably earlier anaesthetic starts. Candidate components include identifying a “Golden Patient” the preceding afternoon to lock in readiness and logistics [15,16]; instituting a standardised 15-minute readiness call from theatre to the holding area or ward to trigger final checks and portering [17]; commencing the multidisciplinary brief on time with named cover for handovers; and ensuring dependable access to a dedicated holding space adjacent to orthopaedic theatres [18]. These measures are low-cost, operationally achievable, and amenable to real-time monitoring.

The study has several practical strengths. It prospectively evaluated four orthopaedic theatres in a high-acuity major trauma centre, targeted a single, clearly defined point in the pathway, and used simple, reproducible metrics (minutes after 08:00 and a prespecified on-time threshold) that are readily interpretable and transferable to other settings. The intervention was deliberately easy to replicate, enabling implementation without altering case mix or staffing, and thereby isolating operational effects. With effective use of the holding bay model, Kagan R et al. have demonstrated that implementing a designated block bay can lead to significant improvements in first-case start times [19].

Limitations also warrant consideration. The short observation window makes this study susceptible to day-to-day variability, such as case load, staff absences, and bed status, which carries the risk of overinterpreting noise as signal. The sample size was modest, yielding wide CIs around estimates for anaesthetic start and on-time performance. Adoption of the holding bay was inconsistent, with only 35 out of 70 recorded cases using the holding bay. The reasons for non-use were not characterised (e.g., capacity constraints, staffing, clinical appropriateness, or preference), introducing selection bias and diluting the observable effect. Team-level factors beyond the intervention, such as overrunning trauma meetings and staff absences, introduced variability in the punctuality of the morning brief. Several co-factors likely influenced start times but were not measured or adjusted for, including porter availability, bed status in the holding bay and wards, theatre staffing and punctuality, case mix and complexity, and concurrent workflow changes (e.g., list order, equipment readiness). The single-centre design and short observation window limit generalisability and preclude assessment of sustainability.

Future work should address these three gaps. Firstly, characterise the reasons for non-use of the holding bay and quantify their frequency (e.g., capacity, staffing, clinical exceptions) so that the bundle can be targeted at residual barriers. Secondly, measure the downstream pathway explicitly, porter response times, handover timing, and team presence at brief. These measures will allow clear insight into where minutes are gained or lost. Thirdly, performance needs to be evaluated and monitored using statistical process control (SPC) over several months to determine the sustainability of these improvements. With consistent use and tighter control of downstream steps, earlier readiness should translate into reliably on-time starts and a higher proportion of Green cases.

## Conclusions

A closely proximate preoperative holding area advanced patient readiness and modestly shifted first-case anaesthetic starts earlier, with an approximately eight-minute improvement in send-for time. Translation to consistently earlier induction was limited by operational constraints, including handover timing, porter response, theatre readiness, and team punctuality, which need to be characterised for further analysis. The findings are also limited in generalisability by the single-centre, pre-post design, small sample size, and major-trauma context. They should therefore be interpreted as exploratory rather than definitive. A pragmatic bundle, comprising pre-identification and pre-assessment of a “Golden Patient,” punctual multidisciplinary briefs with named handover cover, and dependable access to a dedicated holding space, offers a pathway to convert earlier readiness into reliably on-time first-case starts and improved theatre productivity.

## References

[1] (2025). Nottingham University Hospitals NHS Trust. East Midlands Major Trauma Centre (QMC). https://www.nuh.nhs.uk/major-trauma-centre/.

[2] Care Quality Commission (2025). Care Quality Commission. Nottingham University Hospitals NHS Trust (Provider Code RX1): Inspection Reports. https://www.cqc.org.uk/provider/RX1/reports.

[3] Getting It Right First Time (GIRFT (2025). NHS England. Getting It Right First Time (GIRFT) Theatre Productivity Programme. https://gettingitrightfirsttime.co.uk/hvlc/theatre-productivity/.

[4] Ahmed K, Khan N, Anderson D (2013). Introducing the productive operating theatre programme in urology theatre suites. Urol Int.

[5] (2025). NHS England. Improvement guide: theatres, surgery and perioperative care. https://www.england.nhs.uk/publication/improvement-guide-theatres-surgery-and-perioperative-care/.

[6] Allen RW, Taaffe KM, Neilley V, Busby E (2019). First case on-time starts measured by incision on-time and no grace period: a case study of operating room management. J Healthc Manag.

[7] Knox C, Harper J, McMillan L (2024). Increasing first case on time starts in the operating room using an electronic readiness dashboard: a quality improvement project. Perioper Care Oper Room Manag.

[8] Ida JB, Schechter JH, Olmstead J (2025). Addressing late-arriving surgeons in support of first-case on-time starts. Pediatr Qual Saf.

[9] Hicks KB, Glaser K, Scott C, Sparks D, McHenry CR (2020). Enumerating the causes and burden of first case operating room delays. Am J Surg.

[10] Ang WW, Sabharwal S, Johannsson H, Bhattacharya R, Gupte CM (2016). The cost of trauma operating theatre inefficiency. Ann Med Surg (Lond).

[11] Vaughan-Burleigh S, Marsden L, Peagam R (2024). 578 Optimising emergency theatre efficiency with the use of a pre-anaesthetic holding bay, a quality improvement project. British J Surg.

[12] Ogrinc G, Davies L, Goodman D, Batalden P, Davidoff F, Stevens D (2016). SQUIRE 2.0 (Standards for QUality Improvement Reporting Excellence): revised publication guidelines from a detailed consensus process. BMJ Qual Saf.

[13] Firde M, Ayine B, Mekete G, Sisay A, Yetneberk T (2024). Root causes of first-case start time delays for elective surgical procedures: a prospective multicenter observational cohort study in Ethiopia. Patient Saf Surg.

[14] Schliep M, Wu A, Abutaleb A (2021). Analyzing and improving endoscopy unit efficiency in an academic tertiary care facility. Int J Gastroenterol Liver Dis.

[15] Key T, Reid G, Vannet N, Lloyd J, Burckett-St Laurent D (2019). 'Golden Patient': a quality improvement project aiming to improve trauma theatre efficiency in the Royal Gwent Hospital. BMJ Open Qual.

[16] Curley D, Shahmiri A, Arulampalam T (2022). 683 Improving CEPOD efficiency: the ‘Golden Patient’. BJS.

[17] Fletcher D, Edwards D, Tolchard S, Baker R, Berstock J (2017). Improving theatre turnaround time. BMJ Qual Improv Rep.

[18] Saleem J, Brown O, Mclean C, Kurzatkowski K, Radha S, Mallina R (2025). The provision of a trauma bed in theatre recovery and its impact on trauma theatre efficiency: experience from a high-volume trauma unit. Ann R Coll Surg Engl.

[19] Kagan R, Zhao S, Stone A (2020). Spinal anesthesia in a designated block bay for total joint arthroplasty: improving operating room efficiency. Reg Anesth Pain Med.

